# Chronic solvent-induced encephalopathy: course and prognostic factors of neuropsychological functioning

**DOI:** 10.1007/s00420-018-1328-1

**Published:** 2018-06-25

**Authors:** Evelien van Valen, Ellie Wekking, Moniek van Hout, Gert van der Laan, Gerard Hageman, Frank van Dijk, Angela de Boer, Mirjam Sprangers

**Affiliations:** 1 Department of Geriatrics, University Medical Center Utrecht, Utrecht, The Netherlands; 2 Netherlands Center for Occupational Diseases, Coronel Institute of Occupational Health, Amsterdam Public Health Research Institute, Academic Medical Center, Amsterdam, The Netherlands; 3 Mental Health Center Dijk en Duin, Parnassia Groep, Castricum, The Netherlands; 4 Department of Medical Psychology, Medisch Spectrum Twente Hospital, Enschede, The Netherlands; 5 Foundation Learning and Developing Occupational Health (LDOH), Hilversum, The Netherlands; 6 Department of Health Sciences, University of Milano, Milano, Italy; 7 Department of Neurology, Medisch Spectrum Twente Hospital, Enschede, The Netherlands; 8 Coronel Institute of Occupational Health, Amsterdam Public Health Research Institute, Academic Medical Center, Amsterdam, The Netherlands; 9 Medical Psychology, Amsterdam Academic Medical Centers, Amsterdam Public Health Research Institute, Academic Medical Center, Amsterdam, The Netherlands

**Keywords:** Chronic solvent-induced encephalopathy, Follow-up, Course, Prognosis, Neuropsychological assessment, Neurotoxicity, Organic solvents, Diagnostic evaluation

## Abstract

**Purpose:**

Working in conditions with daily exposure to organic solvents for many years can result in a disease known as chronic solvent-induced encephalopathy (CSE). The aims for this study were to describe the neuropsychological course of CSE after first diagnosis and to detect prognostic factors for neuropsychological impairment after diagnosis.

**Methods:**

This prospective study follows a Dutch cohort of CSE patients who were first diagnosed between 2001 and 2011 and underwent a second neuropsychological assessment 1.5–2 years later. Cognitive subdomains were assessed and an overall cognitive impairment score was calculated. Paired *t* tests and multivariate linear regression analyses were performed to describe the neuropsychological course and to obtain prognostic factors for the neuropsychological functioning at follow-up.

**Results:**

There was a significant improvement on neuropsychological subdomains at follow-up, with effect sizes between small and medium (Cohen’s *d* 0.27–0.54) and a significant overall improvement of neuropsychological impairment with a medium effect size (Cohen’s *d* 0.56). Prognostic variables for more neuropsychological impairment at follow-up were a higher level of neuropsychological impairment at diagnosis and having a comorbid diagnosis of a psychiatric disorder at diagnosis.

**Conclusions:**

Results are in line with previous research on the course of CSE, stating that CSE is a non-progressive disease after cessation of exposure. However, during follow-up the percentage patients with permanent work disability pension increased from 14 to 37%. Preventive action is needed in countries where exposure to organic solvents is still high to prevent new cases of CSE.

**Electronic supplementary material:**

The online version of this article (10.1007/s00420-018-1328-1) contains supplementary material, which is available to authorized users.

## Introduction

Organic solvents may cause harm to the nervous system (White and Proctor 1997) by affecting a range of neuronal processes at different levels: changes in ion channel receptors, cellular responses, tissue responses, changes in brain regions and ultimately in cognitive processes and behaviour (Bushnell et al. 2010; van Thriel 2015; Sainio 2015). People are working with organic solvents in industries such as shoe manufacturing, car repair, industrial or house painting, spray painting, furniture manufacturing, printing and cleaning.

A small subsample of workers who are exposed to organic solvents for a long time—e.g. daily exposure for 5 years or more—have been found to develop a syndrome called chronic solvent-induced encephalopathy (CSE). The severity and duration of the solvent exposure play a role in the development of CSE, but there are also individual variations in the susceptibility to the effects of solvent exposure (Godderis et al. 2010; Kezic et al. 2006). Individual differences exist in toxification and detoxification of solvents in the human body (Sainio 2015).

International diagnostic criteria for this syndrome have been described by the World Health Organization (1985), and subsequently refined by a working group in Raleigh (Baker and Seppäläinen 1986), the European Union (European Commission 2009) and a neuropsychological consensus group (van Valen et al. 2012). CSE is recognized as an occupational disease by the International Labour Organization (2010).

The syndrome of CSE is characterized by symptoms of forgetfulness, concentration problems, fatigue, irritability, mood changes and neuropsychological impairment on measures of speed of information processing, speed of motor performance, and immediate memory (van Valen et al. 2012). SPECT scans and fMRI of CSE patients showed decreased dopaminergic activity in the frontostriatal circuitries (Visser et al. 2008) and electroencephalography showed decreased activity in event-related potentials in the posterior parts of the frontoparietal regions (Keski-Säntti et al. 2012). These neurological measures are only found at group level and do not differentiate across individual cases, they cannot (yet) be used for individual diagnostic evaluation. Therefore the diagnostic assessment of CSE relies on neuropsychological assessment for substantiating the neurotoxic effects of organic solvent exposure.

The results found in CSE patients have also been observed in a population of solvent exposed workers; an fMRI study in solvent exposed workers and non-exposed controls has found lower activity in brain regions of the anterior cingulate cortex, the prefrontal cortex and the parietal cortex (Tang et al. 2011). An earlier review of the neuroimaging data on solvent exposed workers, animal data and case reports of people suffering from solvent abuse has yielded contradicting results and no clear dose–response relationships between solvent exposure and neurological changes could be concluded. The poor quality of several of the studies did not allow for further conclusions (Ridgway et al. 2003).

An effective treatment for neuropsychological impairment constituting CSE does not exist (Åbjörnsson et al. 1998; van Hout et al. 2008). Preventive measures against further deterioration of the disease, such as complete cessation or drastic reduction of the exposure, are often advised.

Studies on the neuropsychological disease course of CSE after first diagnosis indicate that neuropsychological functioning of most patients has stabilized or improved at follow-up (Åbjörnsson et al. 1998; Bruhn et al. 1981; Dryson and Ogden 2000; Edling et al. 1990; Lindström et al. 1982; Morrow et al. 1991; Ørbæk and Lindgren 1988). Prognostic factors for the neuropsychological course have been investigated. For patients with more severe neuropsychological deficits at diagnosis there was more improvement at follow-up (Dryson and Ogden 2000). Mixed results were found regarding higher age (Lindström et al. 1982; Ørbæk and Lindgren 1988) and a history of peak exposure as negative prognostic factors (Morrow et al. 1991; Ørbæk and Lindgren 1988). Follow-up studies of CSE patients have been critically reviewed by the ‘Deutsche Gesetzliche Unfallversicherung’ (2007) and van Valen et al. (2009).

These follow-up studies have some limitations. They do not elaborately describe the diagnostic criteria hampering the comparability of the CSE patient groups across studies. The studies employ small sample sizes, ranging from 21 to 86 CSE (type 2 of WHO criteria) patients. Only three studies used multivariate analyses for assessing possible prognostic factors for the neuropsychological course of CSE (Lindström et al. 1982; Morrow et al. 1991; Ørbæk and Lindgren 1988). None of the studies checked for possible problems with performance validity (also called insufficient effort/malingering/non-credible performance) or involvement in litigation. Standardized psychiatric evaluations for comorbid psychopathology were not used. The mean follow-up time varied across studies, ranging from 1.3 to 7 years (van Valen et al. 2009).

The main objectives of this study are to examine the course of the disease and the prognostic factors of neuropsychological functioning of CSE patients, taking these shortcomings into account. The following questions are addressed: (1) what is the course of neuropsychological and psychiatric functioning, and psychological symptoms of CSE patients over time? (2) Which prognostic variables predict the neuropsychological functioning of CSE patients at follow-up? Results of this study can be used to inform patients and other stakeholders about the course of CSE and the prognostic factor(s) that may be subject of intervention in individual patients.

## Methods

### Study design and setting

This prospective study follows a cohort of CSE patients from the Dutch hospitals Academic Medical Center Amsterdam and Medical Spectrum Twente Enschede who are first diagnosed between 2001 and 2011 and have a second neuropsychological assessment 1.5–2 years later. The follow-up assessment of the last patient in this study took place in April 2014.

Clinical data are anonymized. As this study concerns care as usual, no extra interventions or medical and psychological assessments are conducted and no special research approval was needed. Therefore, a dispensation decision for this study was obtained from the Medical Ethics Committee of the Academic Medical Center Amsterdam. The patient care and data handling are according to guidelines of the Dutch legislation, declaration of Helsinki and the local guidelines of the Academic Medical Center and Medical Spectrum Twente.

### Diagnostic procedure

**Fig. 1:**
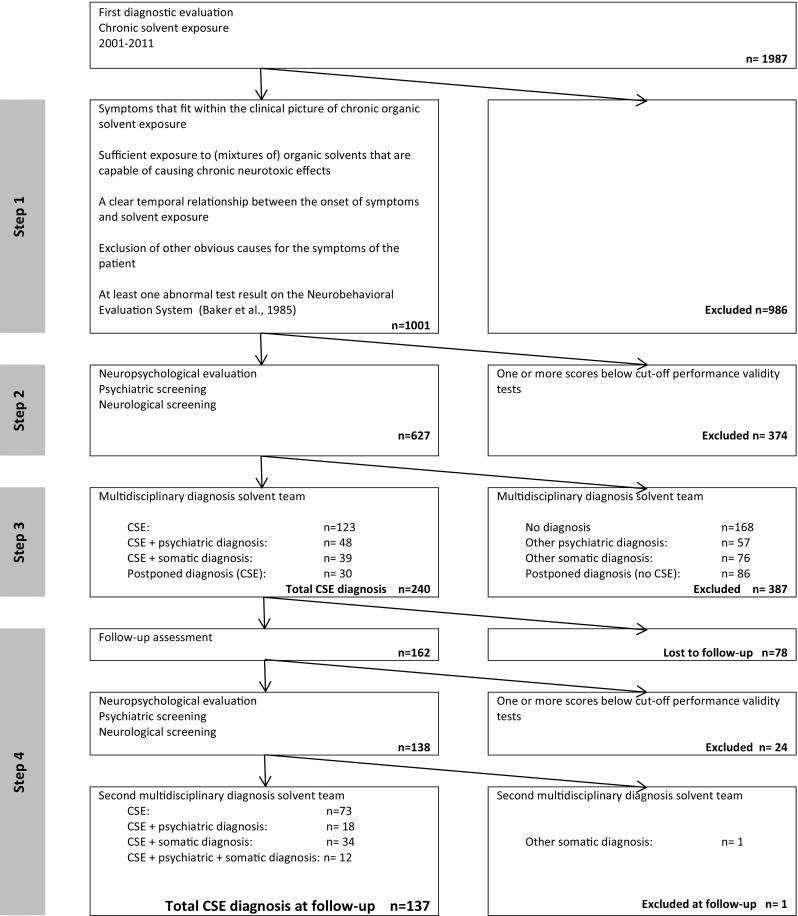
Flow chart referrals Solvent Teams 2001–2011 to patient group selection follow-up

In the Netherlands, there are two specialized centres for the assessment of cases of chronic solvent-induced encephalopathy. These multidisciplinary centres are called Solvent Teams and consist of an occupational physician, occupational hygienist, clinical neuropsychologist, and neurologist. If necessary, a toxicologist, psychiatrist or other medical specialist is consulted. The diagnostic protocol of the Solvent Teams (van der Laan et al. 1995; van Valen et al. 2015) uses a stepwise approach to assess the international consensus-based criteria for CSE (WHO 1985; Baker and Seppäläinen 1986; European Commission 2009; van Valen et al. 2012). The diagnostic steps are represented in the flow chart of Fig. 1.

### Participants

Patients are eligible for inclusion when they are referred to the Dutch Solvent Teams for a first diagnostic evaluation for occupational chronic solvent exposure-related health complaints. Patients who complete the whole diagnostic process and are diagnosed having CSE, are invited for a follow-up assessment. The CSE patients who are re-assessed and still meet the criteria for CSE constitute the patient group of this study.

The included CSE patients are diagnosed according to the diagnostic criteria Type 2 of the WHO (WHO 1985) and Type 2B of the Raleigh criteria (Baker and Seppäläinen 1986), which means that five diagnostic criteria are met: (1) the symptoms fit within the clinical picture of chronic organic solvent exposure. (2) There is exposure for at least 5 years to (mixtures of) organic solvents that are capable of causing chronic neurotoxic effects. (3) There is a clear temporal relationship between the onset of symptoms and solvent exposure. (4) Other major somatic or psychiatric causes for the symptoms of the patient are ruled out. (5) Neuropsychological assessment shows impairments that fit within the cognitive profile of CSE. We added a sixth criterion, because suboptimal performance on neuropsychological assessment is highly prevalent in this patient group (van Hout et al. 2003) and this decreases the diagnostic validity of the neuropsychological assessment. (6) Performance validity of the neuropsychological tests are above cut-off (44 on the third booklet of Test of Memory Malingering, (TOMM, Tombaugh 1996) and 80 on the Amsterdam Short-Term Memory test, (ASTM, Schmand et al. 1999), indicating no problems with mental effort, suboptimal performance or malingering on the neuropsychological assessment.

#### Variables

##### Outcome variables

*Neuropsychological functioning* Neuropsychological functioning is the main outcome of this study. Neuropsychological functioning is composed of the neuropsychological domains memory, attention, motor function and intellectual function. Within the neuropsychological domains, subdomains are configured according to European neuropsychological consensus (van Valen et al. 2012) and included tests are listed in Table 1. Raw scores from the neuropsychological tests at diagnosis and follow-up assessment are compared to normative data of the test manual or in some tests national available updates of the normative data (Schmand et al. 2012) (see Table 1) and are converted to *z* scores.

**Table 1:** Tests of neuropsychological assessment Solvent Team

Domain	Subdomain	Adjustment norm data			Test/norm reference
	Test	Age	Gender	Education	
Memory	Immediate recall				
	NES2 digit span forward	+	+	+	Baker and Letz (1985)
	NES2 digit span backward NES2	+	+	+	Baker et al. (1985)
	Dutch California Verbal Learning Test, total list	+	+	−	Mulder et al. (1996)
	Rivermead stories, immediate recall	+	+	+	Schmand et al. (2012)
	Wechsler memory scale-revised, immediate recall	+	−	−	Wechsler (1987)
	Delayed recall				
	Dutch California Verbal Learning Test, consolidation	+	+	−	Mulder et al. (1996)
	Rivermead stories, delayed recall	+	+	+	Schmand et al. (2012)
	Wechsler memory scale-revised, delayed recall	+	−	−	Wechsler (1987)
	Recognition				
	Dutch California Verbal Learning Test, recognition	+	+	−	Mulder et al. (1996)
	Recognition memory test, faces	+	−	−	Warrington (1984)
Attention	Simple attention				
	NES2 symbol–digit substitution	+	+	+	Baker et al. (1985)
	Trail Making Test, form A	+	−	+	Schmand et al. (2012)
	Stroop test words	+	+	+	Schmand et al. (2012)
	Stroop test colours	+	+	+	Schmand et al. (2012)
	Complex attention				
	NES2 colour–word vigilance	+	+	+	Baker et al. (1985)
	Trail Making Test, form B (relative to A)	+	−	+	Schmand et al. (2012)
	Stroop test colour–word, interference	+	+	+	Schmand et al. (2012)
Motor function	Motor speed				
	NES2 simple reaction time	+	+	−	Baker et al. (1985)
	NES2 finger tapping dominant hand	+	+	+	Baker et al. (1985)
	NES2 finger tapping non-dominant hand	+	+	+	Baker et al. (1985)
	Dexterity				
	NES2 hand–eye coordination	+	+	+	Baker et al. (1985)
	Grooved pegboard, dominant hand	+	−	−	Heaton et al. (1986)
	Grooved pegboard, non-dominant hand	+	−	−	Heaton et al. (1986)
Intellectual function	Verbal function				
	Verbal fluency animals	+	+	+	Schmand et al. (2012)
	Verbal fluency occupations	+	+	+	Schmand et al. (2012)
	WAIS-III similarities	+	+	−	Wechsler (2001, 2004)
	Visuoconstruction				
	WAIS-R/WAIS-III block design	+	+	−	Wechsler (1981, 2001, 2004)
	Rey complex figure test copy	+	−	−	Visser (1970)

*Neuropsychological compound scores subdomains* For the description of the course of neuropsychological functioning, there is a need for data-reduction. The 28 test variables will be reduced to nine compound scores that cover four neuropsychological domains: memory (immediate recall, delayed recall and recognition), attention (simple attention and complex attention), motor function (motor speed and dexterity) and intellectual functioning (verbal intelligence and visuoconstruction). In Table 1 the tests are listed. The compound scores are calculated by averaging the *z* scores within the subdomains. For each subdomain there should be at least two test variables available to calculate a compound score. If two or more test variables of the same test are present within a subdomain (e.g. pegboard dominant hand and pegboard non-dominant hand), they are counted as one score by averaging the *z* scores of the different variables of the specific test.

*Neuropsychological impairment score* A single measure for neuropsychological impairment at diagnosis and follow-up is obtained. The *z* scores of the 28 neuropsychological test variables are aggregated into a total neuropsychological impairment score by summing the number of test scores below − 1.65*z*. In case of missing data, a test score is considered as not impaired. The total neuropsychological impairment score ranges between 0 (no neuropsychological impairment) to 28 (impairment indicated by all test variables of neuropsychological assessment).

*Psychiatric functioning* Psychiatric functioning was measured with a semi-structured clinical interview assessing the axis I disorders of the DSM-IV using the SCID-I (van Groenestijn et al. 1999; First et al. 1996). For each psychiatric diagnostic category of the SCID-I, a dichotomized score is obtained: 0—no diagnosis and 1—criteria for psychiatric disorder are met. A dichotomized score for overall axis I psychopathology is used with 0 indicating no psychopathology and 1, one or more psychiatric axis I disorders according to DSM-IV criteria.

In case of a psychiatric diagnosis on the SCID-I in the domain of mood or anxiety disorders, a clinician-rated measure of the severity of the psychiatric impairment is obtained using the Hamilton rating Scale for Depression (HRSD) (Hamilton 1960) or Hamilton Anxiety Rating Scale (HARS) (Hamilton 1959). The HRSD consists of 21 items, of which 17 contribute to the total score. Eight items have a scale ranging from 0 (absent) to 4 (severe), 9 items have a scale ranging from 0 (absent) to 2 (obviously present). The total score ranges from 0 to 52 with 0–7 labelled as no depression, 8–13 as mild, 14–18 mild to moderate, 19–27 moderate to severe and > 28 severe. The HARS consists of 14 items which are scored on a scale ranging from 0 (absent) to 4 (severe). The total score ranges from 0 to 56, with 0–17 labelled as mild, 18–24 as mild to moderate, 25–30 moderate to severe and > 30 severe anxiety problems.

*Psychological symptoms* The severity of psychological symptoms is measured with the Symptom Check List (SCL-90), which includes 90 items that are combined to form 9 subscales and a total psychological symptoms score. A 1–5 response scale is employed with the total score ranging between 90 and 450. Normative data of different patient groups and the general population of the Netherlands are available (Derogatis 1975; Ettema and Arrindell 2003).

##### Predictors

*Age* Age is measured in years at time of the first neuropsychological assessment (diagnosis) and at time of the follow-up assessment.

*Education* For education the Dutch Verhage score is used, with seven categories: ranging from unfinished primary school to university degree (Verhage 1964).

*Exposure* Cumulative exposure is estimated in retrospect with the formula described in the “Appendix”. The exposure estimation is based on the information obtained from the patient, in combination with the knowledge on solvent use in diverse sectors at different times in the Netherlands (Fransman et al. 2002). In the rare case of the availability of air monitoring measurements or biomonitoring data, these are also incorporated in the estimation of the cumulative exposure. The range of the cumulative exposure estimation is between 0 for no exposure up to more than 400 in extreme cases. The number of years of working while exposed is recorded. This is a variable not including the weighing of the severity of the exposure. This measure is used for comparability purposes with other studies.

The number of months since the last exposure is listed. And a variable is computed whether exposure has ceased, diminished or is still ongoing at time of diagnosis and follow-up.

*Somatic comorbidity* The presence of somatic diseases is enquired during the interview, retrieved from medical files or diagnosed by the neurologist and the occupational physician. A total score for somatic diseases with partly similar symptoms as CSE is obtained by summing the following conditions: cardiac problems, hypertension or hypotension, diabetes, hypothyroidism, neurological disease (including sleep disorders and polyneuropathies), migraine, and respiratory problems (including asthma and allergies). The total score is dichotomized into 0: no somatic comorbidity and 1: at least one somatic comorbidity.

*Alcohol* Use of alcohol is measured in units of alcoholic drinks per week. The amount of alcoholic consumptions is recalculated to “standard glasses” in which 1 unit represents a maximum of 12 ml or 10 g of alcohol within the drink (for example, 1 unit is a beer of 250 ml, 1.3 units represents a bottle of beer, 1 unit of strong liquor is 35 ml, and 1 unit of wine is 100 ml).

*Use of psychotropic medication* Use of psychotropic medication is dichotomized into 0, no psychotropic medication versus 1, use of psychotropic medication (e.g. selective serotonin reuptake inhibitors, selective noradrenaline reuptake inhibitors, tricyclic antidepressants, benzodiazepines, antipsychotics, anti-epileptics and opioid painkillers).

*Work situation* Whether a patient still works in his or her occupation is recorded. We distinguish different categories of work situation: currently working in the same job where most of the exposure was obtained; currently working but in another job than where most of the exposure was obtained; on sick leave for at least 2 weeks; not working but receiving disability pension, retirement or unemployment. For the analysis the variable is dichotomized into: currently working (1) and not-working (0).

*Litigation* Involvement in litigation process is dichotomized as 0—no involvement and 1—involvement, such as legal procedures against former employer or procedures against social security agencies or private insurance companies.

##### Background variables

*Occupation* Occupation is operationalized as the occupation related to exposure to solvents, with the following classes of occupations: painters, spray-painters, printers, chemical workers or people working in the paint industry, floor layers, upholsterers and other occupations (e.g. shipyard workers and shoe factory workers).

*Follow-up time* Follow-up time is measured in number of months between the first and the second neuropsychological assessment.

### Statistical analysis

To describe possible selection bias, the study group is compared to the ‘lost to follow-up’ group and the ‘group with below cut-off performance validity on the follow-up assessment’ on demographic characteristics, neuropsychological and psychiatric functioning at first diagnostic assessment, using oneway ANOVA and post hoc tests (LSD correction) or Kruskal–Wallis tests for non-parametric variables.

The course of neuropsychological and psychiatric functioning and psychological symptoms over time is tested with paired *t* tests or for non-parametric variables, with the *χ*^2^ test. Cohen’s *d*s are calculated for the effect size of the paired *t* tests (an effect size is labelled small if *d*: 0.2, medium if *d*: 0.5 and large if *d*: 0.8 or higher).

For the identification of possible prognostic factors a linear regression analysis is performed, with the total neuropsychological impairment score at follow-up as the dependent variable.

Possible prognostic factors at diagnosis include neuropsychological impairment at first diagnostic assessment, psychiatric functioning, psychological symptoms, age, education, cumulative exposure index, a history of peak exposure, somatic comorbidity, units of alcohol per week, use of psychotropic medication, working situation and involvement in litigation. All prognostic factors are evaluated with univariate analyses. Only factors with *p* < 0.20 are selected for the multivariate linear regression. Subsequently, a check for multicollinearity is performed using scores of variance inflation factors and in case of positive results the collinear factor is excluded.

## Results

### Participants

From 2001 to 2011, 1,987 patients have been referred to the Solvent Teams for a first diagnostic evaluation for chronic solvent exposure-related health complaints. Most patients were referred by general practitioners (51%), followed by referrals by occupational physicians (22%), neurologists (11%) and other medical specialists (16%). At the time of the first diagnostic step most patients were still working (47%) or on short-term sick leave (22%). Others received disability pension (21%), were unemployed (6%) or retired (4%).

The inclusion flow chart is presented in Fig. 1. 1,001 patients met the inclusion criteria 1–4 of the WHO. Of these, 627 patients showed sufficient performance validity on the TOMM and ASTM and could proceed in the diagnostic procedure. 37% of the patients from step 1 were excluded in step 2 due to insufficient performance validity. The multidisciplinary team subsequently identified 240 patients with a CSE diagnosis or a combined differential diagnosis with CSE.

After first diagnosis, 78 CSE patients were lost to follow-up. The most common reasons were: the long traveling distance to one of the Solvent Teams, the effort and energy it would take to undergo a neuropsychological assessment, the wish to end the sometimes difficult diagnostic process and diagnosis, the wish to avoid the confrontation with one’s disabilities, and the possible negative impact of a new diagnostic evaluation on the status of one’s disability pension or other kind of financial compensation.

The ‘lost to follow-up’ group (*n* = 78) and the patients who were excluded at follow-up due to insufficient performance validity (*n* = 24) did not differ from the study group with respect to age (*F*(2,236) = 0.79, *p* = 0.46), education (*F*(2,236) = 2.53, *p* = 0.08) and work situation (*χ*^2^(2, *N* = 235) = 0.37, *p* = 0.83). However, significant differences were found between groups with respect to involvement in a litigation procedure (*χ*^2^(2, *N* = 238) = 7.74, *p* = 0.02) with percentages of patients involved in a litigation procedure ranging from 15% in the study group to 29% in the lost to follow-up group and 38% in the insufficient performance validity group. At time of first diagnosis, no significant differences were found on number of impaired tests at neuropsychological assessment between the study group, the lost to follow-up group and the decreased performance validity at follow-up group (*F*(2,235) = 0.72, *p* = 0.49). The groups also did not differ with regard to the severity of psychological symptoms (*F*(2,232) = 0.05, *p* = 0.95).

### Descriptive data

The characteristics of the study participants are listed in Table 2. There are only 3 (2%) women in the study group. At time of first diagnosis most of the participants are in their 40s and 50s (80%) and most have a history of more than 15 years of daily solvent exposure (83%). More than half of the patients have ceased or lowered exposure prior to the first diagnostic assessment. Most of the study participants are blue collar workers with lower occupational education (71%). At time of follow-up the majority of patients is not exposed to solvents anymore (75%). Also, at time of follow-up many patients have a different working situation. The percentage of patients needing some sort of social security (sick leave or long-term disability pension) had increased from 43 to 55%. Among these, the percentage patients with a permanent work disability pension increased noticeably from 14 to 37%. The somatic comorbidity at diagnosis and follow-up is listed in Table 3.

**Table 2:** Demographic data (*n* = 137)

	At diagnosis		At follow-up	
	Frequency	%	Frequency	%
Gender				
Male	134	98	–	–
Female	3	2	–	–
Age				
< 30 years	3	2	3	2
31–40 years	20	15	12	9
41–50 years	53	38	51	37
51–60 years	58	42	63	46
61–70 years	3	2	8	6
Education				
Primary education	7	5	–	–
Lower occupational (no diploma)	17	13	–	–
Lower occupational (diploma)	73	53	–	–
Mid-level	37	27	–	–
College and above	3	2	–	–
Exposure level				
Low (cumulative index < 15)	9	6	–	–
Intermediate (cumulative index 15–49)	33	25	–	–
High (cumulative index ≥ 50)	95	69	–	–
Exposure years				
0–5 years	0	0	–	–
6–15 years	23	17	–	–
16–20 years	27	19	–	–
> 20 years	87	64	–	–
Current exposure				
Ongoing exposure	35	25	10	7
Exposure decreased	30	22	25	18
Cessation of exposure	72	53	103	75
Occupation				
Painters	47	35	–	–
Spray-painters	39	28	–	–
Printers	19	14	–	–
Chemical/paint industry	5	4	–	–
Floor layers	3	2	–	–
Upholsterers	8	6	–	–
Other	16	11	–	–
Work situation				
Working, same job	65	48	28	20
Working, other job	12	9	34	25
Sick leave	35	25	14	10
Disability pension	20	14	50	37
Retired	1	1	4	3
Unemployed/welfare	4	3	7	5
Litigation				
Yes	20	15	–	–
No	117	85	–	–
Alcohol use				
No alcohol	48	35	46	33
1–7 per week	51	38	63	46
8–14 per week	18	13	15	11
15–28 per week	14	10	9	7
> 28 per week	6	4	4	3
Solvent Team diagnosis				
CSE	65	47	73	53
CSE and psychiatric diagnosis	32	22	18	13
CSE and somatic diagnosis	24	18	34	25
CSE and psychiatric and somatic diagnosis	18	13	12	9
Time between first diagnosis and follow-up assessment				
Mean number of months (sd; median)			21	(14; 21)
			(Min 6	Max 88)

– Means no change from diagnosis to follow-up

**Table 3:** Somatic comorbidity

	At diagnosis	At follow-up
	*n*	*n*
Total somatic diseases	42 (30%)	46 (34%)
Cardiac problems	8	10
Hypertension	16	16
Hypotension	4	2
Diabetes type 2	11	12
Hypothyroidism	3	3
Neurological diseases	7	7
Migraine	6	7
Respiratory problems	12	14

### Course of neuropsychological functioning

For each test variable a percentage of impaired tests results at group level is given reflecting the percentage of tests scores below − 1.65*z*, see Table 4. The most impaired test results are found in the subdomains immediate recall of memory, simple and complex attention and motor speed. In the domain of intellectual functioning few patients had impaired test results; 1–17% impairments.

**Table 4:** Neuropsychological functioning

Domain	Subdomain test	At diagnosis				At follow-up				Cohen’s *d*	*n*
		Mean (sd)	*Z* score (sd)	*n*	% impaired	Mean (sd)	*Z* score (sd)	*n*	% impaired		
Memory	Immediate recall		− 0.8 (0.5)	137			− 0.6 (0.6)	135		0.38	135*
	NES2 digit span forward	5.5 (0.7)	− 0.9 (0.8)	137	12	5.7 (0.8)	− 0.6 (0.8)	126	6		126
	NES2 digit span backward NES2	4.8 (0.8)	− 0.9 (0.7)	137	10	5.1 (0.8)	− 0.6 (0.7)	126	4		126
	Dutch California Verbal Learning Test, total list	40 (8.0)	− 1.3 (0.9)	137	29	41 (9.7)	− 1.1 (1.0)	137	26		137
	Rivermead stories, immediate recall	15 (5.2)	− 1.0 (1.0)	137	30	15 (4.8)	− 1.0 (0.9)	136	25		136
	Wechsler memory scale-revised, immediate recall	31 (5.6)	0.0 (1.0)	136	2	33 (5.8)	0.4 (1.1)	135	2		134
	Delayed recall		− 0.6 (0.7)	137			− 0.3 (0.6)	136		0.37	136*
	Dutch California Verbal Learning Test, consolidation	33 (10.2)	− 0.6 (1.2)	137	18	37 (11.9)	− 0.3 (1.2)	137	11		137
	Rivermead stories, delayed recall	11 (4.8)	− 0.8 (0.9)	136	16	12 (4.8)	− 0.7 (0.9)	134	13		133
	Wechsler memory scale-revised, delayed recall	26 (8.5)	− 0.3 (1.1)	136	6	28 (7.5)	0.1 (1.0)	135	3		134
	Recognition		− 0.6 (0.8)	137			− 0.6 (0.9)	137		0.04	137
	Dutch California Verbal Learning Test, recognition	38 (3.6)	0.1 (1.0)	137	5	39 (3.6)	− 0.1 (1.2)	137	12		137
	Recognition memory test, faces	39 (4.8)	− 1.3 (1.3)	137	40	40 (5.4)	− 1.0 (1.5)	137	31		137
Attention	Simple attention		− 1.3 (0.8)	137			− 1.1 (0.9)	137		0.54	137*
	NES2 symbol–digit substitution	3.2 (0.9)	− 1.1 (1.2)	137	35	3.0 (0.8)	− 0.8 (1.2)	131	23		131
	Trail Making Test, form A (s)	46 (16)	− 1.0 (1.1)	137	28	42 (15)	− 0.7 (1.1)	137	18		137
	Stroop test words (s)	60 (14)	− 2.0 (1.0)	137	66	60 (16)	− 1.9 (1.1)	136	60		136
	Stroop test colours (s)	77 (16)	− 1.7 (0.9)	137	52	75 (20)	− 1.5 (1.2)	136	49		136
	Complex attention		− 0.9 (0.5)	137			− 0.7 (0.6)	137		0.44	137*
	NES2 colour–word vigilance (ms)	828 (135)	− 1.9 (0.8)	137	64	779 (150)	− 1.4 (1.0)	132	42		132
	Trail Making Test, form B (relative to A) (s)	118 (45)	− 0.7 (1.0)	133	16	115 (64)	− 0.6 (1.2)	136	18		133
	Stroop test colour–word (interference) (s)	132 (36)	− 0.3 (0.7)	137	3	125 (43)	− 0.0 (0.7)	135	2		135
Motor function	Motor speed		− 1.5 (0.7)	137			− 1.2 (0.8)	131		0.39	131*
	NES2 simple reaction time, msec	412 (126)	− 2.1 (0.5)	137	83	389 (128)	− 1.9 (0.8)	132	66		132
	NES2 finger tapping dominant hand	142 (45)	− 0.9 (1.2)	137	29	154 (40)	− 0.5 (1.1)	131	14		131
	NES2 finger tapping non-dominant hand	137 (43)	− 0.8 (1.3)	137	28	153 (55)	− 0.4 (1.3)	131	15		131
	Dexterity		− 0.5 (1.3)	136			− 0.3 (1.3)	117		0.15	116
	NES2 hand–eye coordination	1.7 (0.8)	0.6 (1.1)	137	3	1.5 (0.4)	0.7 (1.1)	130	2		130
	Grooved pegboard, dominant hand (s)	83 (24)	− 2.0 (2.7)	136	43	81 (18)	− 1.4 (1.7)	124	31		123
	Grooved pegboard, non-dominant hand (s)	86 (23)	− 1.3 (2.0)	136	27	88 (37)	− 1.2 (2.1)	124	20		123
Intellectual function	Verbal function		− 0.8 (0.7)	136			− 0.6 (0.6)	120		0.27	120*
	Verbal fluency animals	19 (5)	− 0.8 (0.9)	137	15	19 (6)	− 0.7 (1.1)	132	17		132
	Verbal fluency occupations	14 (4)	− 0.7 (0.9)	137	12	14 (4)	− 0.5 (1.0)	132	11		132
	WAIS-III similarities	20 (6)	− 0.8 (0.9)	136	12	21 (5)	− 0.6 (0.9)	121	7		121
	Visuoconstruction		− 0.4 (0.7)	134			− 0.4 (0.8)	109		0.00	107
	WAIS-R block design	26 (11)	0.1 (1.0)	82	2	28 (11)	0.4 (1.0)	48	1		48
	WAIS-III block design	29 (13)	− 0.3 (0.9)	54	3	27 (13)	− 0.5 (1.0)	64	6		40
	Rey complex figure test copy, faults	9 (7)	− 0.7 (1.0)	134	15	8 (6)	− 0.6 (1.0)	131	10	0.1 (1.2)	129

% impaired is the percentage of patients scoring on a specific test variable below − 1.65 z

*Paired sample *t* tests sign at *p* < 0.01

Paired sample *t* tests of the mean *z* scores showed significant improvement at follow-up on 6 of the 9 subdomains. The size of the improvement at group level ranges between 0.1 and 0.3 *z* scores.

The mean neuropsychological impairment of patients at diagnosis (7.2, sd 3.3) and at follow-up (5.5, sd 3.3), decreased significantly (*t*(136) = 6.5, *p* = 0.000) with a medium effect size of Cohen’s *d* 0.56.

### Course of psychiatric functioning and psychological symptoms

In Table 5, psychiatric diagnoses and corresponding mean Hamilton mood and anxiety severity ratings are given. The semi-structured psychiatric interview was found to be indicative of a DSM-IV psychiatric axis I disorder in 49 of the 137 included patients at time of diagnosis. At follow-up, the number of patients with psychiatric axis I diagnoses decreased to 30 (*χ*^2^(1, *N* = 137) = 19.6, *p* = 0.000).

**Table 5:** Psychiatric functioning

	At diagnosis		At follow-up	
	*n*		*n*	
Psychiatric axis I disorders	49	(36%)	30	(22%)
		Mean Hamilton Depression (sd)		Mean Hamilton Depression (sd)
Mood disorders+	39	18.2 (6.1)	19*	20.0 (3.3)
Major depression	28		15	
Dysthymia	6		3	
Depression NOS	5		1	
Bipolar depression	0		0	
Psychosis	0		0	
		Mean Hamilton Anxiety (sd)		Mean Hamilton Anxiety (sd)
Anxiety disorders++	24	17.2 (6.6)	19*	18.7 (7.2)
Agoraphobia	3		0	
Panic disorder	4		7	
Panic disorder with agoraphobia	2		2	
Social phobia	8		5	
Specific phobia	1		1	
Generalized anxiety disorder	8		6	
Post-traumatic stress disorder	0		0	
Hypochondria	0		0	
Obsessive compulsive disorder	1		0	
Substance abuse	13		13	
Alcohol abuse lifetime	11		11	
Alcohol addiction lifetime	2		2	
Drug abuse lifetime	3		3	
Drug addiction lifetime	0		0	
Drug abuse current	0		0	
Current abstinence from alcohol	3		2	
Current alcohol use	10	12.3 per week (16.7)	11	8.1 per week (10.0)
Other psychiatric disorders				
Somatic disorder	0		0	
Somatoform disorder	0		0	
Pain disorder	1		0	
Eating disorder	0		0	

*Chi-square tests of number of mood and anxiety disorders at follow-up *p* < 0.05

The total score of the psychological symptoms of the study group have significantly decreased at follow-up; from a mean total score at diagnosis of 185 (sd = 57) to a mean total score of 173 (sd = 51); *t*(134) = 3.6, *p* = 0.000 with a small effect size of Cohen’s *d* 0.31. The mean level of psychological symptoms would be labelled by the normative data of the SCL-90 as very high at first diagnosis and high at follow-up in comparison with a norm group of age and gender matched general population (Ettema and Arrindell 2003).

### Prognostic variables at diagnosis for neuropsychological impairment at follow-up

Univariate linear regression analyses identified six possible prognostic factors measured at time of diagnosis for inclusion in the multivariate regression model for the neuropsychological impairment at follow-up: neuropsychological impairment, psychiatric functioning, psychological symptoms, education, weekly alcohol intake and the use of psychotropic medication, see Table 6. There was no indication of multicollinearity. A statistically significant multivariate linear regression model was obtained (*F*(6,130) = 14.89, *p* < 0.000) with a *R*^2^ of 0.407. Severity of neuropsychological impairment and psychiatric functioning (a comorbid axis I psychiatric disorder) at diagnosis remained significant predictive factors for a relatively high level of neuropsychological impairment at follow-up.

**Table 6:** Prognostic factors for level of neuropsychological impairment at follow-up

Variables measured at diagnosis	Univariate		Multivariate	
	*B*	Sig.	*B*	Sig.
(Constant)			3.933	0.011
Neuropsychological impairment	0.593	0.000	0.558	0.000
Psychiatric functioning	1.301	0.025	1.083	0.041
Psychological symptoms	0.011	0.022	− 0.004	0.390
Age	0.015	0.662		
Education	− 0.968	0.003	− 0.476	0.080
Cumulative exposure	0.003	0.464		
History of peak exposure	0.109	0.898		
Somatic comorbidity	− 0.668	0.270		
Weekly alcohol intake	− 0.061	0.047	− 0.032	0.185
Use of psychotropic medication	0.968	0.120	0.280	0.586
Work situation	− 0.663	0.239		
Involvement in litigation	0.184	0.813		

## Discussion

The current study aims to contribute to the understanding of the (neuro)psychological course and predictors of CSE by studying a cohort of CSE patients over an average of 21 months after their first diagnosis, in the Netherlands. The neuropsychological impairment in CSE patients was found to decrease over time, although the magnitude of the reduction was medium to small. The psychological symptoms have improved over time and fewer patients meet the DSM-IV criteria for psychopathology (a decrease from 36 to 22%). At time of follow-up however, neuropsychological impairment and psychological problems still exist. The percentage patients with a work disability pension increased noticeably during follow-up.

Neuropsychological impairment at diagnosis was found to predict the level of neuropsychological impairment at follow-up. Moreover, a psychiatric DSM-IV diagnosis at time of CSE diagnosis was also found to predict neuropsychological impairment at follow-up.

These results are in line with previous research on the course of CSE, stating that CSE is a non-progressive disease with no severe deterioration after cessation of exposure (van Valen et al. 2009).

Cumulative exposure to organic solvents and peak exposure did not predict the course of the neuropsychological functioning of CSE patients. This contradicts an earlier study which has found peak exposure as a predictor of the neuropsychological course (Morrow et al. 1991) although Ørbæk and Lindgren (1988) also did not find a significant effect of exposure on the neuropsychological test performance at follow-up in a prognostic study. In the current study, there is no evidence for a dose–response relationship between exposure and the course of the neuropsychological functioning in CSE patients. The design of a longitudinal follow-up study on the heavily selected patient population of diagnosed CSE patients is not adequate to obtain knowledge about dose–response relationships. An inclusion criterion for a CSE diagnosis is a cumulative exposure high enough to be able to cause CSE. The patients in this study therefore all have sufficient exposure to be able to cause adverse health effects. As a consequence, the majority of the patients in this study only do vary in range of cumulative exposure between intermediate and high, only 6% of the patients has a low exposure corresponding with at least 6 years of solvent exposure and a maximum of 44 years. For evidence for (the absence of) a dose–response relationship there is a need for cohort studies in the original population of workers in which the solvent-exposed workers are compared to a well-chosen population of workers with no exposure. Another reason to be very cautious using a prognostic design for etiological conclusions is that the course of an occupational disease after diagnosis may not be influenced by the previous exposure.

The long-term effects of chronic solvent exposure in workers after cessation of exposure have also been described in epidemiological cohort studies and follow-up studies of exposed workers and unexposed controls. These point in the direction of a high lifetime exposure to organic solvents causing more psychological problems and an exacerbation of age-related cognitive impairment (Dick et al. 2010; Nordling Nilson et al. 2002, 2003, 2007, 2010; Sabbath et al. 2014). The results are in agreement with the hypothesis that exposure to organic solvents in working life decreases the cognitive reserve capacity (Stern 2002) and thereby causes an acceleration of the normal aging process, even many years after exposure has ceased. In our study we did not find such aging processes progressing over time. To the contrary, instead of deterioration we found an improvement of neuropsychological functioning over 2 years. We do not know whether the cognitive aging effects as found in epidemiological studies would be found in our CSE patients when the follow-up time would be longer, at least 10 years. However, a matched control group is needed for this kind of study to distinguish between effects of ‘pure’ aging and late effects of solvent exposure.

Leaving the exposure to solvents as a causal explanation for the neuropsychological impairment of CSE-patients out of consideration, one could state that the CSE-patients identified at first diagnosis are in fact patients with non-amnestic mild cognitive impairment (MCI) (Petersen 2016; Gerstenecker and Mast 2014). Mild cognitive impairment is found in the aging population. Patients show no signs (yet) of interference in activities of daily life, but do have mild cognitive impairment at neuropsychological assessment. These MCI-patients mimic the neuropsychological profile of CSE-patients evaluated at one measurement in time. However, the disease course of MCI differs from CSE. Although two meta-analyses found evidence for a reversion of neuropsychological deficits in 18–24% of MCI-patients (Canevelli et al. 2016; Malek-Ahmadi 2016), most studies found an increase of neuropsychological impairment and a progression from MCI to dementia with prevalences depending on the subtype of MCI (Amieva et al. 2004; Petersen 2016; Aerts et al. 2017). Patients with MCI who are assessed with a number of the same neuropsychological tests and compared to the same normative data as the CSE patients in this study, show comparable sizes of neuropsychological impairment at first diagnosis (Schmand et al. 2014), although it must be noted that this MCI study group is older than our CSE-patient group. After 2 years, the MCI-group shows a mean deterioration of 0.6 sd, which differs from our finding of an overall improvement in neuropsychological functioning of CSE patients. Also, within 2 years 23% of their entire study group and 41% of the patients diagnosed with MCI at first diagnostic evaluation progresses into dementia, compared with none of the CSE-patients in our study group progressed into dementia within the short follow-up period. In conclusion: the neuropsychological course of CSE differs from the course of MCI and indicates other underlying aetiology.

This is the first follow-up study of CSE-patients that included a classification of psychiatric disorders according to DSM-IV criteria. When looking at the similar level of psychological symptoms in the previous follow-up studies, it seems likely that these studies must have included CSE-patients who met the criteria of a psychiatric disorder. Comorbid psychopathology in our CSE-patients is quite frequent (36% at diagnosis, 22% at follow-up) and psychopathology at diagnosis is a negative prognostic factor for the course of CSE. As there are several evidence-based treatment options for common mental disorders such as anxiety and mood disorders, motivating newly diagnosed CSE patients for psychiatric treatment should be of priority. There is no evidence-based treatment for CSE, but many patients could benefit from treatment of their comorbid psychiatric disorder and improve their quality of life as well as possibly also their neuropsychological prognosis. However, some caution must be taken into account: evidence-based guidelines for common mental disorders are constructed for patients with psychopathology only. In other patient groups with a combination of a somatic disease and a psychiatric disorder psychopharmaceutic treatment or psychotherapies have been shown to be not as effective as for patients without somatic comorbidity (e.g. Kootker et al. 2017; Tedeschini et al. 2011). As half of the patients in this study group need some kind of social security and probably have loss of income (Eshuis 2010), it can be inferred that a part of these patients has decreased social contacts and daily activities, taking into account that the large majority is between 40 and 60 years old. The decrease in purposeful, social and pleasurable activities is a risk factor for psychopathology and could be target of rehabilitation interventions or contextual psychological interventions such as behavioural activation (Richards et al. 2016). As there is only a little evidence base for the treatment options of this CSE-patient group, the intervention choices for the consequences of CSE in daily life will be an educated guess, using scientific data from other patient groups.

Some limitations of this study merit attention. A major limitation is the lack of a control group. This has been partly covered by the use of adequate normative data, but these norm groups differ across neuropsychological tests. An advantage of the different normative data is the spread of control data across norm populations. Another limitation is the retrospective measurement of cumulative exposure, rendering the exposure estimation less reliable, which might explain the lack of significant results related to exposure data. The number of statistical analyses in relation to the number of people in the study group is fairly high. Some significant findings might be due to chance, although effect sizes point in the direction of small-to-medium effects. Whereas more study patients would have been desirable, CSE is a rare disease and we chose to apply stringent diagnostic criteria, further reducing the number of patients within the study group. A limitation of the design of the study is that the diagnosis of CSE at follow-up was not blind. Only one patient was excluded at follow-up because of evidence for a neurodegenerative disease (Parkinson). Blinding of the previous diagnosis of the patients, or diagnosis by another team, could have changed the diagnosis, although for every patient the diagnostic criteria were applied in a protocolled manner. In practice, it was not possible to set up another diagnostic team for the blinding of the diagnosis at follow-up.

This study also has several strengths. In the Netherlands, there is a unique curative infrastructure for the multidisciplinary diagnosis of CSE. The two Solvent Teams are established and rather well-known centres of expertise and patients are referred from all over the country for diagnosis. The diagnosis is made on medical grounds and the Solvent Teams do not have a role in financial compensation procedures. The diagnostic assessment is covered by the mandatory health insurance for the Dutch population. This means that the Solvent Teams have a national coverage of the total number of CSE patients. Furthermore, the protocolled diagnostic procedure is well described and is the same for all included patients. Patients are only selected at time of first diagnosis and are prospectively followed up after diagnosis. The patient selection is well described and only showed differences between lost to follow-up and patient group with respect to involvement in litigation. The study group was less involved in litigation, which decreases the influence of secondary gains on the description of the course of CSE. With respect to the neuropsychological assessment, patients with concerns regarding performance validity are excluded and psychopathology is classified according to DSM-IV criteria. Also, this is the first follow-up study in which all previously found predictors are measured and analysed within one patient group.

Future studies would benefit from longer follow-up times, enabling the investigation of whether CSE patients remain relatively stable, although a longer follow-up period in a clinical patient group has the risk of increased loss to follow-up and the risk of a biased study group selection. A longer follow-up period on the other hand could give more insight if there is a part of the patient group who do progress into dementia or have an exacerbation of age-related cognitive impairment.

Future studies of newly diagnosed patients however will not likely take place in the Netherlands; the number of new CSE cases is declining, due to improved working conditions and outsourcing labour in jobs with high solvent exposure to low and middle-income countries where regulation and hygienic conditions are less stringent. So the problem of adverse health effects of chronic solvent exposure is also outsourced to other countries. It is important that knowledge on the harmful effects of solvent exposure is available in the countries where most of the exposure is nowadays. Preventive measures such as the use of water-based paints and glues and personal protection measures should be of priority to prevent the incidence of CSE globally. The good news is that by substitution and by organizing safe working conditions, CSE is a disease that can be prevented, so action is needed. In countries with diminishing incidences of CSE, vigilance is needed to keep working conditions safe and to prevent new cases of CSE.

## Electronic supplementary material

Below is the link to the electronic supplementary material.

Supplementary material 1 (DOCX 15 KB)
