# Supplementary material for: Chronic solvent-induced encephalopathy: course and prognostic factors of neuropsychological functioning
Source: Int Arch Occup Environ Health. 2018 Jun 25;91(7):843–58. doi: 10.1007/s00420-018-1328-1 (PMC6132664; doi:10.1007/s00420-018-1328-1)
Supplement: Supplementary file 1 — Supplementary material 1 (DOCX 15 KB) [file 420_2018_1328_MOESM1_ESM.docx]

**Appendix 1: cumulative exposure index**

The cumulative exposure index is calculated by an occupational hygienist considering the following formula: a*b*c*d*e*f.

In this formula,

**a** stands for adjusted years of exposure (years working on the job adjusted for the weekly hours of exposure. For example, for a job of 40 hours per week with fulltime exposure, the years working in the job were multiplied with 0.4.

**b** stands for the Occupational Air Requirement (OAR) which represents the quantity of air (m3) required to dilute the vapour concentration in the work room resulting from 1 L product to a concentration below the Occupational Exposure Limit (OEL) (Brouwer et al. 2005; Zock et al; 1998; [www.schoentechniekveilig.nl](http://www.schoentechniekveilig.nl) 2006). (1= low, 3= moderate, 5= high).

**c** stands for application method of the solvents in relation to time of application (1= small surface, for example using a brush, 2= average surface, for example using a roller, 4= large surface, for example spray painting and/or working in high temperatures).

**d** stands for frequency of peak exposure 1= no peak exposure, 1.5= some peak exposure, 2= frequent peak exposures),

**e** stands for ventilation (1= working indoors without ventilation, 0.8= working indoors with natural ventilation, for example with an open window, 0.6= working indoors with source extraction, 0.4= mostly working outdoors)

**f** stands for the use of personal protective equipment (1= no personal protective equipment or inadequate personal protective equipment, 0.75= use of personal protective equipment).

When patients had different occupations the formula was calculated for each job and the scores were summarized into one cumulative exposure index.

The total score describing the severity of exposure employs three categories: 0-15 low, 15-50 intermediate, >50 high lifetime cumulative exposure. These categories are used for clinical communication purposes, but not for the statistical analysis in this study.

A history of peak exposure is element **d** of the cumulative exposure index.

**Appendix 2: 3 short vignettes to illustrate the exposure index:**

**Anthony** is a 42 year old man who has worked as a painter for 24 years. He has been self-employed and has always used solvent-rich paints during his career. He did not follow new legislation on the use of water-based paints indoors until he developed health complaints recently. He has worked fulltime since he was 18 years old. During the spring and summer season he worked outdoors and in the fall and winter he used to work indoors, painting walls, windows and stairs as a subcontractor in in newly developed houses. For degreasing metal constructions, like elevators or metal door frames he used trichloroethylene. He used to wash his hands in turpentine after the work day. He has never used personal protection devices. He does not report peak exposure incidences in which he has felt drowsy, nausea or light-headed.

**a**: (0.4*24=) 9.6, **b**: 3, **c**: 1, **d**: 1, **e**: 0.4, **f:** 1= 11.5 index of cumulative exposure

**Bart** is a 50 years old man who used to work at a cheese factory until he was 34 years old. He was not exposed to solvents during this job. When the factory closed, he decided to change his career path and retrained as a painter in the yacht industry. He had worked as a yacht painter for 10 years, where he used to paint new constructed yachts in a warehouse with some natural ventilation. He reports to have felt regularly high of the solvents in the paint, he was often light-headed when returning home from work and he needed a walk about once a week during the work day because he felt dizzy and nausea of the exposure to solvents in the warehouse. There were several colleagues working in the same hall with glass fiber reinforced polyester and exposure to styrene and acetone. He figured that he was quite good at his job and he chose to develop his spray painting skills. He became a furniture spray painter at a local workshop nearby for 6 years. He used to work in a warm booth, with some, but inadequate exhaust ventilation and did not use personal safety measures according to regulations. The carbon filters of his masks were not changed regularly and often he did not use the mask at all: he did not like the way the mask fitted his face. Also, at the workshop other colleagues made fun of him when he used the mask.

**a**: (0.4*10=) 4.0, **b**: 1, **c**: 2, **d**: 1.5, **e**: 0.8, **f:** 1= 9.6 index of cumulative exposure

**a**: (0.4*6=) 2.4, **b**: 3, **c**: 4, **d**: 1.5, **e**: 0.8, **f:** 1= 34.6 index of cumulative exposure

total: 44 index of cumulative exposure

**Chris** is an upholsterer who has been for 40 year in the trade. He used to lay carpets on floors and staircases. He used solvent rich glues containing toluene and Methyl Ethyl Ketone and always worked indoors. There was never professional ventilation as he worked in the private homes of clients. He reports frequent instances of peak exposure. He did not use personal protective equipment.

**a**: (0.4*40=) 16, **b**: 5, **c**: 2, **d**: 1.5, **e**: 1, **f:** 1= 240 index of cumulative exposure

Brouwer D, de Pater N, Zomer C, Lurvink M, van Hemmen JJ. An experimental study to investigate the feasibility to classify paints according to neurotoxicological risks: Occupational Air Requirement (OAR) and indoor use of alkyd paints. Ann. Occup. Hyg, 2005.;49(5):443-451.

Zock JP., Stouten Th J, van Hemmen JJ. Occupational Air Requirement (OAR) and [In Dutch: Occupational Air Requirement (OAR) en vervanging van oplosmiddelen voor verf en verfproducten]. 1998; Rapport V98.1241, TNO Voeding, Zeist

<http://www.schoentechniekveilig.nl/media/gezondheidscode_v2.pdf>; TNO Quality of Life, September 2006
